# Evaluation of the impact of the COVID-19 lockdown on the quality of life of patients monitored for cancer who practice an adapted physical activity: rugby for health

**DOI:** 10.1007/s00432-021-03621-7

**Published:** 2021-04-05

**Authors:** Stéphanie Motton, Kelig Vergriete, Luc Nguyen VanPhi, Eric Lambaudie, Audrey Berthoumieu, Jean Pous, Martine Delannes, Julien Piscione, Caroline Cornou, Benoit Bataille, Diane Saxod, Fabien Pillard

**Affiliations:** 1 Department of Surgical Oncology, Institut Universitaire du Cancer Toulouse-Oncopole, Toulouse, France; 2 Department of Surgical Oncology, Referring Physician of the RUBieS Marseille Division of Rugby Club Stade Phocéen, Institut Paoli Calmettes, Marseille, France; 3 Referring Physician of the RUBieS Parisis Division of Parisis Rugby Club, Parisis, France; 4 Occitan Rugby League Medical Committee, Toulouse, France; 5 Research Department, French Rugby Union Federation, Marcoussis, France; 6 Department of Surgical Oncology, Referring Physician of the RUBieS Clermont-Ferrand Division, Association Sportive Montferrandaise Clermont Auvergne, Centre Jean Perrin, Clermont-Ferrand, France; 7 Intensive Care Department, Hôpital de Narbonne, Narbonne, France; 8 Centre Hospitalier Universitaire Toulouse, Hospital Pierre Paul Riquet, University Sports Clinic, Toulouse, France

**Keywords:** Physical activity, Cancer, Quality of life, Psychological impact, Rugby for health

## Abstract

**Purpose:**

The benefits of regular physical exercise on the tolerability of cancer treatments, quality of life and survival rates post-diagnosis have been demonstrated but all supervised physical activities have been interrupted due to the global health crisis and the need for lockdown to halt the spread of SARS-CoV-2. To reintroduce activities post-lockdown, we wanted to assess the impact of the COVID-19 lockdown on the quality of life and the psychological status of patients who practice an adapted physical activity such as rugby for health.

**Methods:**

The evaluation was conducted in two phases: an initial self-questionnaire comprised of 42 questions sent to all participants to assess the impact of lockdown and a second assessment phase in the presence of the participants. We assessed anthropometric data, functional fitness parameters, quality of life and the psychosocial status of the subjects. The data were compared to pre-lockdown data as part of a standardised follow-up procedure for patients enrolled in the programme.

**Results:**

105/120 (87.5%) individuals responded to the rapid post-lockdown survey analysis. In 20% of the cases, the patients reported anxiety, pain, a decline in fitness and a significant impact on the tolerability of cancer treatments. Twenty-seven patients agreed to participate in the individual analysis. Following lockdown, there was a significant decrease in the intensity of physical activity (*p* = 8.223e–05). No post-lockdown changes were noted in the assessments that focus on the quality of life and the level of psychological distress. Conversely, there was a significant correlation between the total of high energy expended during lockdown and the quality of life (*p* = 0.03; rho = 0.2248) and the level of psychological distress post-lockdown (*p* = 0.05; rho = − 0.3772).

**Conclusion:**

Lockdown and reduced physical activity, particularly leisure activities, did not impact the overall health of the patients. However, there was a significant correlation with the level of physical activity since the higher the level of physical activity, the better the quality of life and the lower the level of psychological distress.

**Supplementary Information:**

The online version contains supplementary material available at 10.1007/s00432-021-03621-7.

## Introduction

In the midst of the emergence of the concept of “health sports” and the implementation of several specific interventions for patients over the past few years, a new coronavirus (COVID-19) pandemic was declared to be a global health emergency by the World Health Organisation (WHO) on 11 March 2020. Due to the high contagiousness and aggressiveness of the disease in certain subjects, particularly the elderly and those with co-morbidities, many countries had to enact a lockdown to slow the spread of the virus. In fact, when this article was being written, no vaccine was available and only barrier measures such as distancing, masking and hand washing were effective. In France, for example, the government decided to restrict people to their homes from 14 March until 11 May. Only people with a profession indispensable to life or a reason related to a requirement for care were allowed to leave home (Jee 2020; Ghanchi 2020). Obviously, although this lockdown was necessary, it had negative psychological and physical effects on individuals, the full consequences of which only time will reveal. One of the main effects was the reduction of physical activity and consequently the effects of a sedentary lifestyle (Furtado et al. 2021; Odone et al. 2020; Colizzi et al. 2020; Avancini et al. 2020a). In recent years, it has become clear that supervised adapted physical activity (APA) plays an important role in rehabilitation for cancer survivors. Overall, meta-analyses of the most common cancers show that weekly physical activity of minimal intensity and duration improve quality of life, the tolerability of cancer treatments and survival rates (Rossi et al. 2015; Ibrahim and Al-Homaidh 2011; Schmid and Leitzmann 2014). In fact, life after cancer remains a real physical and psychological ordeal, marked by a deterioration in the quality of life for one-third of the patients. Persistent fatigue and an increase in anxiety and depression are frequently reported. Physical sequelae resulting in functional limitations are also observed in one out of two individuals (Rossi et al. 2015).

In this context, many sports federations have worked alongside medical experts to develop appropriate forms of their sport for patients with chronic diseases, including cancer. The indications and contraindications of these activities are set out in a new standard drawn up by the French Olympic Committee, called *Médico Sport Santé *(Santé and par le CNOSF 2017)

Since January 2016, the French Rugby Federation (FFR) has made the “*Rugby à 5 Santé*” (R5S—rugby for health) programme available. This is an adapted non-contact activity that can be prescribed as part of a tertiary prevention programme for patients with chronic diseases such as cancer. This activity does not involve competition. Since November 2017, the *Rugby Union Bien Être et Santé* (RUBieS) association, in collaboration with the FFR, has endorsed a quality label for the care, medical follow-up and assessment of cancer patients participating in the R5S activity.

Field training stopped abruptly with the first lockdown. Recent studies have already pointed out the negative impact of physical inactivity caused by the social isolation imposed by the public health authorities due to COVID-19 (Rossi et al. 2015; Ibrahim and Al-Homaidh 2011). However, no observational study on any group of participants who are cancer survivors has been conducted.

Our aim was to assess the real impact of abrupt cessation of physical activity on cancer survivors during the lockdown by analysing R5S players. We considered this study to be important to reorganise the resumption of activity and to suggest measures to increase physical activity in cancer survivors during the COVID-19 pandemic.

## Materials and methods

### Participants

To participate in the study, participants had to have been diagnosed with and monitored for cancer. They also had to be a member of a RUBieS-labelled division. All RUBieS-labelled divisions offer the same type of training based on a 10-min warm-up, group workshops to assimilate the rules of 5-a-side rugby for health, improve technical skills, play group games (40 min) and at the end of the session, do stretching exercises (10 min).

The participants were required to provide their consent as well as a certificate of approval to participate from their oncologist and sport physician.

### Procedures (Fig. 1)

**Fig. 1:**
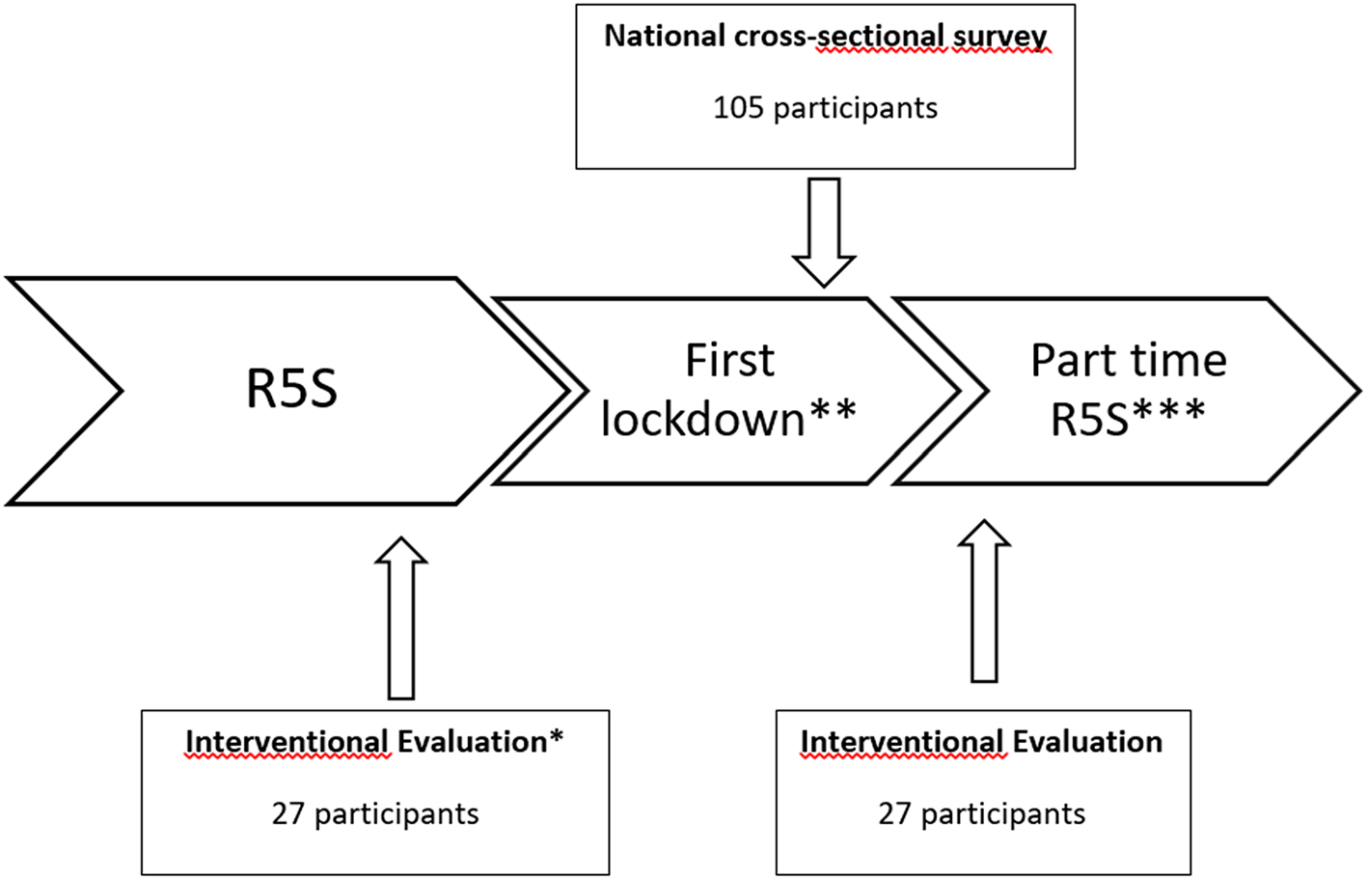
Flow-chart describing the participant recruitment and both procedures. *Interventional evaluation every 3 months, last evaluation before the first lockdown. **From 14 March to 11 May 2020. ***Resumption of 5-a-side rugby only in the Toulouse, Lavaur and Dijon centres

The evaluation was carried out in two stages and by two different procedures.

The first stage consisted of a national cross-sectional survey during the last 7 days of the French lockdown on the experience of participants during this last week. All RUBieS players in France were approached via an internet platform dedicated to the management of medical data and which satisfies all related digital data security obligations.

During this investigation, the subjects completed a self-questionnaire to assess the lockdown [Appendix 1].

The feasibility of the questionnaire was tested beforehand by a sample cohort of 10 patients. After completing the questionnaire, the patients were asked to answer 5 additional questions:

- Did you answer all 42 questions?
- Did you take less than 10 min to answer all of the questions?
- Did you find the questions unambiguous? [*quantification of the answer using a visual analogue scale of 0–10 (VAS 0–10)]*
- Were the questions easy to understand? (*quantification of the answer using a VAS of 0–10)*
- How many questions posed a problem?

The second step was an interventional evaluation of the impact of the lockdown on anthropometric parameters, functional fitness, the quality of life, psychological status and the level of physical activity. In fact, before the lockdown, R5S players were routinely assessed every 3 months as part of their cancer follow-up. The last assessment before the lockdown was in February 2020 (which we considered as the baseline). As soon as the lockdown occurred, after having obtained consent from the patients, we proposed an additional evaluation during the first week post-lockdown (which we considered as the follow-up). This was an individual assessment, identical to those carried out before the lockdown, based on anthropometric measurements, functional fitness tests and self-questionnaires to assess the quality of life, psychological distress and physical activity level. Only participants from 3 divisions who were able to return to activity at the time of the evaluation were asked to participate in the individual evaluations.

### Measures

#### National cross-sectional survey

For the survey, the questionnaire assessed the level of sleep disturbances, anxiety levels, general health and treatment tolerability on a VAS scale of 0–10. According to the literature, a score of 0–3 indicates zero or minimum feelings, 4–6 moderate feelings and 7–10 strong to very strong feelings (Kahl and Cleland 2005).

The 42 questions included in the questionnaire were approved jointly by oncologists and general practitioners acting either as referring physicians for the Rugby Santé programme or who belonged to the FFR Medical Commission (Appendix 1). As this situation is unprecedented and the literature rather sparse, we did not find a validated questionnaire.

#### Interventional evaluation

The second phase was carried out in the presence of the participants. During this phase, we assessed anthropometric data (weight, height, waist and hip circumference), functional fitness parameters, quality of life and the subjects’ psychosocial status. The data recorded were compared against pre-lockdown data (baseline data) as part of a standardised follow-up procedure for patients participating in the R5S programme.

The following functional fitness parameters were assessed in an individual interview with a sports instructor. Interviewers were specially trained in the administration of the performance measurements used in this study.

We chose tests that are easy to perform, reproducible and widely used in clinical research:

- A unipedal stance test was performed according to the following procedure: the participant stood on the foot of their choice. The heel of the opposite foot was placed on the inside of the knee of the supporting leg, eyes kept open and arms held relaxed along the sides of the body. The assessor started timing when the participant was in position. The timer was stopped as soon as the patient lost their balance, started to move, or when the foot was no longer in contact with the knee. The result was recorded in seconds within a maximum time frame of 60 s. Two tests were conducted and the highest value was recorded (Springer et al. 2007).
- The sit-and-reach test: to measure the flexibility of the trunk and the posterior muscle group of the lower limbs. No equipment was required. The participant stood with legs straight, trunk bent and hands stretched as low as possible. Knees could not be bent and the back had to be quite rounded for reaching down. To straighten up, the knees had to be bent. The evaluator observed the participant’s limit when the legs were straight and evaluated the distance between the hands and the ground according to the assessment grid (Mayorga-Vega et al. 2014).
- Shoulder flexibility was assessed by a simple test. The participant stood with their back against the wall, feet slightly apart, 45 cm from the wall. They were required to keep their buttocks, back and shoulder blades pressed against the wall with elbows and wrists stretched upwards against the wall. The assessor measured the distance between the hands and the wall (Springer et al. 2007).
- To evaluate lower limb muscle strength, participants performed the 30-s chair stand test, which is a reliable and valid measurement of lower body strength amongst high-functioning older adults. Participants began in a seated position on a chair 43 cm high. With arms across their chest, they stood up completely and sat down again as many times as possible for 30 s. The best of two tests was used for analysis (Bohannon 1995; Rossi et al. 2016).
- Aerobic fitness was assessed by a 6-min walk test [6MWT]. This time-based test is ideally conducted in a quiet, enclosed corridor. Patients are instructed to walk from one end to the other, covering as much ground as possible in the allotted time period. The distance walked in the specified time period is recorded. It is considered as an objective measurement that provides a means of monitoring response to treatment. The 6MWT is easy to administer, better tolerated, and more reflective of activities of daily living than the other walk tests. Therefore, the 6MWT is currently the test of choice to assess functional walking for clinical or research purposes. In the cancer population, it is considered a valid and reliable cardiopulmonary test (Schmidt et al. 2013; Rossi et al. 2016).

The quality of life (QoL) assessment was carried out using the EORTC QLQ-C30 self-questionnaire (version 3.0) (Aaronson et al. 1993). This questionnaire is used to assess the quality of life of cancer patients. It assesses five functions [Physical functioning (PF), Role functioning (RF), Emotional functioning (EF), Cognitive functioning (CF) and Social functioning (SF)), nine symptoms (fatigue, nausea, pain, dyspnoea, insomnia, loss of appetite, constipation, diarrhoea, and financial difficulties), and the overall general health of patients from cancer diagnosis onwards (Aaronson et al. 1993). Completed questionnaires were returned within 14 days. The following referenced scoring procedure was used. All the scales and single-item measures range in score from 0 to 100. A high score represents a higher response level. Therefore, a high score for a functional scale represents a high/healthy level of functioning, a high score for the global health status/QoL represents a high QoL, but a high score for a symptom scale/item represents a high level of symptomatology. We calculated the raw score (RS) for each item and then used the linear function and coefficients as explained in the EORTC version 3.0 manual (Aaronson et al. 1993).

The level of psychological distress was assessed with the KESSLER K6 self-questionnaire. This questionnaire is a simple scale for measuring the level of psychological distress. The scale comprises six questions on emotional status, to be answered on a scale of 0 (never) to 4 (all the time). The results of the six questions are then added together to give a total score ranging from 0 to 24. A low score indicates a low level of psychological distress, while a high score indicates a high level of psychological distress (Kessler et al. 2002). Completed questionnaires were returned within 14 days.

The level of physical activity was assessed using the GPAQ (Global Physical Activity Questionnaire) (Bull et al. 2009). The GPAQ was developed in 2002 by the WHO as part of the Health Surveillance Programme (STEPS). Results are interpreted by classifying subjects according to the energy expended in METs (metabolic equivalents) per minute and per week according to the intensity of reported and coded physical activity. The calculation of the physical activity level in METs takes into account the time spent on physical exercise in a typical week, the number of days of physical activity and the intensity of the physical activity. Populations were classified according to three levels of physical activity: low, moderate and high. The ranking criteria for each level of physical activity are as follows:

- High-level physical activity: intense physical activity, at least 3 days a week, resulting in energy expenditure of at least 1500 MET min/week OR at least every day walking and moderate or intense physical activity up to a minimum of 3000 MET min/week.
- Moderate-level physical activity: physical activity characterised by at least 20 min of intense physical activity per day for 3 or more days per week OR at least 30 min of moderate physical exercise or walking per day for 5 or more days per week OR at least 5 days of walking and moderate or intense physical activity, up to a minimum of 600 MET min/week, with the level of physical activity remaining below that corresponding to a high level of activity.
- Low-level physical activity: individuals who do not meet any of the afore-mentioned criteria are classified in this category.

### Statistical methods

Percentages were calculated to describe the categorical variables and we present continuous variables as a median with extreme values. We compared categorical and continuous variables between groups using the *χ*^2^ and Mann–Whitney tests, respectively. Box-plots were used to graphically represent the quantitative variables. The Mann–Whitney–Wilcoxon *U* test for continuous variables was chosen to compare the pre- and post-lockdown distributions (baseline and follow-up). Spearman’s rank correlation coefficient was calculated for all parameters and the GPAQ score. We performed all tests using R software version 3.6.3 (https://www.r-project.org) and established the level of significance at *p* < 0.05.

## Results

### Results of the national cross-sectional survey

#### Feasibility of the questionnaire

On the sample cohort of 10 patients tested:

In response to the question “have you answered all 42 questions asked?”, 8 patients answered “yes”. In the 2 cases where the participants answered “no”, they had answered none of the 42 questions.

In response to the question “Did you take less than 10 min to answer all the questions?”, 7 participants answered “yes”, in the other 3 cases, they had taken about 2 min longer.

In response to the question “Did you find the questions unambiguous? (On a scale of 0 to 10)”, 4 subjects answered “0”, 4 people answered “1”, i.e. very mildly ambiguous, and 2 subjects answered “2”, i.e. slightly ambiguous. In all cases, the participants concluded that ambiguity was zero to low.

In response to the question “Were the questions easy to understand? (On a scale of 0 to 10)”, 8 participants answered “0”, i.e. easy to understand and 2 answered “2”, i.e. slightly difficult to understand.

In response to the question “How many questions posed a problem? (between 0 and 42)”, 4 participants said none, 4 said 1 question and 2 said 2 questions.

Seven rugby for health divisions participated in the study. 120 patients were approached and 105 answered the questionnaire between the 7th and the 11th of May 2020, i.e. a participation rate of 87.5%.

In 94% of the cases, the questionnaire was completed. The average time to complete a questionnaire was 6 min and 34 s.

#### Characteristics of the participants (Table 1)

**Table 1:** National cross-sectional survey—characteristics of the participants

Multiple choice answers	Responses	
Under 30 years	5.71%	6
Between 30 and 60 years	71.43%	75
Over 60 years	22.86%	24
Female	89.52%	94
Male	10.48%	11
Smokers no	90.48%	95
Smokers yes	9.52%	10
Breast cancers	66.28%	57
Pelvic cancers	11.63%	10
Abdominal cancers	4.65%	4
Leukaemia	3.49%	3
Prostate cancers	1.16%	1
Kidney cancers	0.00%	0
Lung cancers	1.16%	1
Other cancers	11.63%	10
Surgery	75.58%	65
Chemotherapy	66.28%	57
Radiotherapy	70.93%	61
Hormone therapy	46.51%	40
Radiofrequency	1.16%	1
Other treatments	17.44%	15
Relapse no	89.53%	77
Relapse yes	10.47%	9
Negative COVID-19 Pcr	96.12%	99
Positive COVID-19 Pcr-Asymptomatic	2.91%	3
Positive COVID-19 Pcr with hospitalisation	0.97%	1
Positive COVID-19 Pcr with intensive care admission	0.00%	0

Of the 105 participants, 70% were between 30 and 60 years of age, only 5% were under 30 years of age.

Ninety-four participants were women, i.e. 90% of the cohort.

Only 10% of the respondents were smokers.

In two-thirds of the cases, these were breast cancer follow-up patients. Gynaecological, gastrointestinal and lung cancers in conjunction with malignant blood disorders were represented in the remaining third. In more than 70% of the cases, cancer had been diagnosed within the 5 year-period prior to lockdown.

In 80% of the cases, the participants had at least one of the following cancer treatments: surgery, chemotherapy, radiotherapy or hormone therapy; treatment was ongoing in approximately 40% of the cases. One-third of the cases involved hormone therapy. In 12% of the cases, the participants were in relapse.

Of the 105 participants, 4 reported SARS-CoV-2 infection, 1 of whom required hospitalisation on a regular ward. None of the participants reported a severe form requiring ICU admission.

With regards to physical activity, all the participants practised R5S and two-thirds of the patients had started during the current season. In one-third of the cases, participants had at least two training sessions per week. In almost 80% of the cases, the individuals practised at least one other sport before and in addition to rugby for health. These were mainly running, cycling, walking and swimming. Of the 10% of smokers, approximately 10% had stopped smoking on enrolling for rugby for health.

#### Evaluation of the impact of lockdown on physical activity (Table 2)

**Table 2:** National cross-sectional survey—evaluation of the impact of lockdown on physical activity

Multiple choice answers	Responses	
Since the health crisis, have you continued to practice a physical activity at home?		
Yes	75.00%	78
No	25.00%	26
How many times a week?		
0	17.71%	17
1 to 2 times	34.38%	33
3 to 4 times	20.83%	20
Every day of the week (at least 5 times)	27.08%	26
How long does an average session last?		
0	15.63%	15
Approximately 15 min	8.33%	8
Approximately 30 min	40.63%	39
1 h or more	35.42%	34
Since the health crisis, how long do you walk per day?		
Less than 30 min	42.72%	44
More than 30 min	41.75%	43
More than an hour	15.53%	16
In your opinion, since the beginning of the health crisis, has your energy expenditure increased or decreased?		
Increased	14.42%	15
Decreased	64.42%	67
Stable	21.15%	22

After 56 days of lockdown, 75% of the participants reported continuing physical activity at home such as walking, cycling, gymnastics and yoga. In one-third of the cases, the participants practised this activity once or twice a week and in 20% of the cases up to 5 times a week. In 80% of the cases, the sessions lasted more than 30 min. In two-thirds of the cases, the participants walked for at least 30 min a day. However, two-thirds of the patients noted a decrease in their daily energy expenditure and one-third reported a weight gain of less than 5 kg. Four participants started to smoke again during the lockdown.

#### Evaluation of the impact of the lockdown on behaviour (Table 3)

**Table 3:** National cross-sectional survey—evaluation of the impact of lockdown on behaviour

Multiple choice answers	Responses	
During the health crisis, did you work?		
Yes, at the workplace	22.12%	23
Yes, I teleworked	23.08%	24
No	59.62%	62
Before the crisis, how much time per day did you spend in front of a screen (smartphone, tablet, TV, etc.)		
Less than 3 h	71.15%	74
3 to 6 h	19.23%	20
More than 6 h	9.62%	10
Since the crisis, how much time per day do you spend in front of a screen (smartphone, tablet, TV, etc.)?		
Less than 3 h	42.86%	45
3 to 6 h	39.05%	41
More than 6 h	18.10%	19
Since the lockdown, have you kept in touch with other rugby health players?		
No	9.80%	10
Yes, by phone from time to time	45.10%	46
Yes, very often by phone	25.49%	26
Yes, by video calls from time to time	20.59%	21
Yes, very often by video calls	7.84%	8

During the lockdown, two-thirds of the participants did not work. Before the health crisis, fewer than 20% of the participants reported spending 3–6 h a day in front of screens (tablet, computer, smart phone, and TV) compared to 40% during the lockdown. In 90% of the cases, participants kept in touch with other participants, either by telephone (70%) or by video calls (20%). Contact was frequent in one-third of the cases.

#### Evaluation of the symptoms (Table 4)

**Table 4:** National cross-sectional survey—evaluation of the symptoms

	Sleep disorders (%)	Anxiety (%)	Joint or musculoskeletal pain (%)	Decrease in well-being (%)	Impact on treatment tolerance (%)	Impact on future (%)
0	29.81	24.76	32.69	26.92	40.70	17.82
1	6.73	5.71	5.77	6.73	6.98	5.94
2	7.69	9.52	5.69	4.81	4.65	7.92
3	7.69	16.19	10.58	9.62	3.49	11.88
4	4.81	6.67	5.77	12.50	9.30	5.94
5	16.35	13.33	11.54	15.35	10.47	17.82
6	7.69	3.81	4.81	6.73	2.33	6.93
7	11.54	9.52	15.38	7.69	10.47	4.95
8	3.85	5.71	3.85	6.73	3.49	13.86
9	0.96	0.95	0	0.96	0	1.98
10	2.88	3.81	1.92	0.96	8.14	4.95
M	3.41	3.49	3.20	3.43	3.13	4.25

*M* weighted mean

During the lockdown, half of the participants reported a moderate to severe sleep disorder. One in five participants, i.e. 20%, had severe sleep difficulties.

Approximately 45% of the patients reported moderate to severe anxiety. Approximately 20% experienced very severe anxiety.

Joint and musculoskeletal pain: 43% of the participants reported the onset of new moderate to severe pain during the lockdown. Approximately 20% felt very severe pain.

Fitness assessment: just over 50% of the participants reported a moderate to severe decrease in fitness or in well-being. Just over 15% of the participants noticed a highly significant decline.

The impact of an interruption in activity on treatment tolerability: 45% of the participants reported a moderate to severe impact. Just over 20% deemed the impact to be highly significant.

The future impact of an interruption in activity on overall health: 56% predicted a moderate to severe impact and one in five individuals, i.e. 20%, predicted a very severe impact.

Resumption of the regular physical activities, adapted rugby and/or other activities: 95% of the participants expressed a desire to resume activities. 5% did not know and none gave a negative response.

We analysed the group of participants who experienced a significant or highly significant impact on treatment tolerability following the cessation of activity (7–10). There were 19 patients in this group.

37% of the patients in this group experienced new severe to very severe pain (7–10). Anxiety was also highest in this group, with more than 50% of the participants experiencing severe anxiety (7–10) and significant sleep disorders (7–10).

### Interventional evaluation

#### Characteristics of the participants (Table 5)

**Table 5:** Interventional evaluation—characteristics of the patients

	Number of patients included in the analysis	Percentage of the overall number of patients observed
Females	26	96%
Males	1	4%
Age indicators	55 [27–73]	–
Disease		
Breast cancer	17	63%
Ovarian cancer	3	11%
Cervical cancer	2	7%
Endometrial cancer	1	4%
Other types of cancer	4	15%
Treatment during the study		
Hormone therapy	8	30%
Targeted therapy	1	4%
Chemotherapy	2	7%
Marital status		
Single	10	37%
With partner	16	59%
Widow/widower	1	4%
Professional status		
On sick leave	8	30%
Employed	12	44%
Retired	6	22%
No profession	1	4%

Fifty patients agreed to participate in the study and met the inclusion criteria. Twenty-seven patients from 3 sites participated in all the physical tests and completed all the questionnaires: 63% of the participants came from the Toulouse site, 22% from Dijon and 15% from Lavaur.

The median age of the participants was 55 years and the range was 27–73 years.

96% of the participants were female.

62.9% of the participants had been diagnosed with breast cancer, 22.1% with pelvic cancer and 14.8% with another form of cancer or blood disorder (leukaemia, prostate, and lung). 30% of the participants were receiving hormone therapy. Two participants were undergoing chemotherapy. One person was undergoing targeted therapy.

In terms of marital status, 37% of the subjects were single, 59.3% had a partner and 3.7% were widowed.

Most of the patients were actively employed: 48.2%, 29.6% on sick leave and 11.1% retired.

#### Physical assessment

o Anthropometric measurements and functional fitness test assessment (Table 6):

**Table 6:** Anthropometric data and physical tests before and after lockdown

	Baseline (pre-lockdown)	Follow-up (post-lockdown)	*p *value
Anthropometric data			
Weight	67 [45–106]	69.6 [44–107.9]	**0.007556**
BMI	24.7 [16.5–41.4]	26.16 [16.2–42.1]	**0.01485**
Waist circumference	86 [58–122]	88.5 [62–115]	0.1652
Hip circumference	97 [76–139]	99 [82–134]	**0.04454**
Waist/hip circumference ratio	0.92 [0.75–1.23]	0.89 [0.75–1.17]	0.9153
Physical tests			
Aerobic fitness (6-min walk test)	675 [450–850]	667 [512–812]	**0.03384**
30-s chair stand test	27 [11–40]	24 [14–41]	**0.01408**
Standing balance test	60 [3–60]	60 [3–60]	0.1422
Sit-and-reach test	3 [1–5]	3 [1–5]	0.1236
Right shoulder flexibility	5 [1–5]	5 [1–5]	0.5214
Left shoulder flexibility	4 [1–5]	4 [1–5]	0.6153
Right side muscle strength	39 [23–68]	38 [23–55]	0.3189
Left side muscle strength	38 [24–72]	37 [0–59]	0.4113

Significant values appear in bold

A statistically significant weight gain of 2.6 kg was recorded post-lockdown (*p* = 0.007). Increases in Body Mass Index (BMI) and hip circumference were also statistically significant (*p* = 0.01 and *p* = 0.04, respectively).

After lockdown, there was a significant decrease in aerobic fitness (*p* = 0.03) and a decrease in lower limb muscle strength (*p* = 0.01).

o Assessment of physical activity levels (Table 7):

**Table 7:** Physical level of activity before and after lockdown

	Baseline	Follow-up	*p *value
Total MET expended/week (min)	2400 [480–6480]	720 [0–5280]	**8.223e–05**
MET expended at work/week	0 [0–4800]	0 [0–4800]	0.4203
MET expended for leisure activities/week	1440 [0–4800]	240 [0–1800]	**2.98e–08**
Higher total activity level			
Number of patients observed	10	1	
Percentage	37%	4%	
Mean total activity			
Number of patients observed	12	14	
Percentage	44%	52%	
Low total activity level			
Number of patients observed	5	12	
Percentage	19%	44%	
Sitting or lying down			
Minimum	1	3	
Maximum	12	12	
Mean	4.72	6.81	
Median	4	7	

Significant values appear in bold

Post-lockdown, there was a significant decrease in the intensity of physical activity from 2400 to 720 MET min/week (*p* = 8.223e–05).

Furthermore, less energy was expended in terms of physical activity at work, in daily life, commuting (cycling or walking) and leisure activities during the lockdown period, with a significant decrease in the number of subjects classified as highly active (*p* = 0.003) and a significant increase in the number of subjects with a low activity level (*p* = 0.02).

The same applies to the number of hours spent sitting or lying down, which increased significantly during the lockdown (*p* = 0.03).

#### Primary objective: quality of life evaluation

**Fig. 2:**
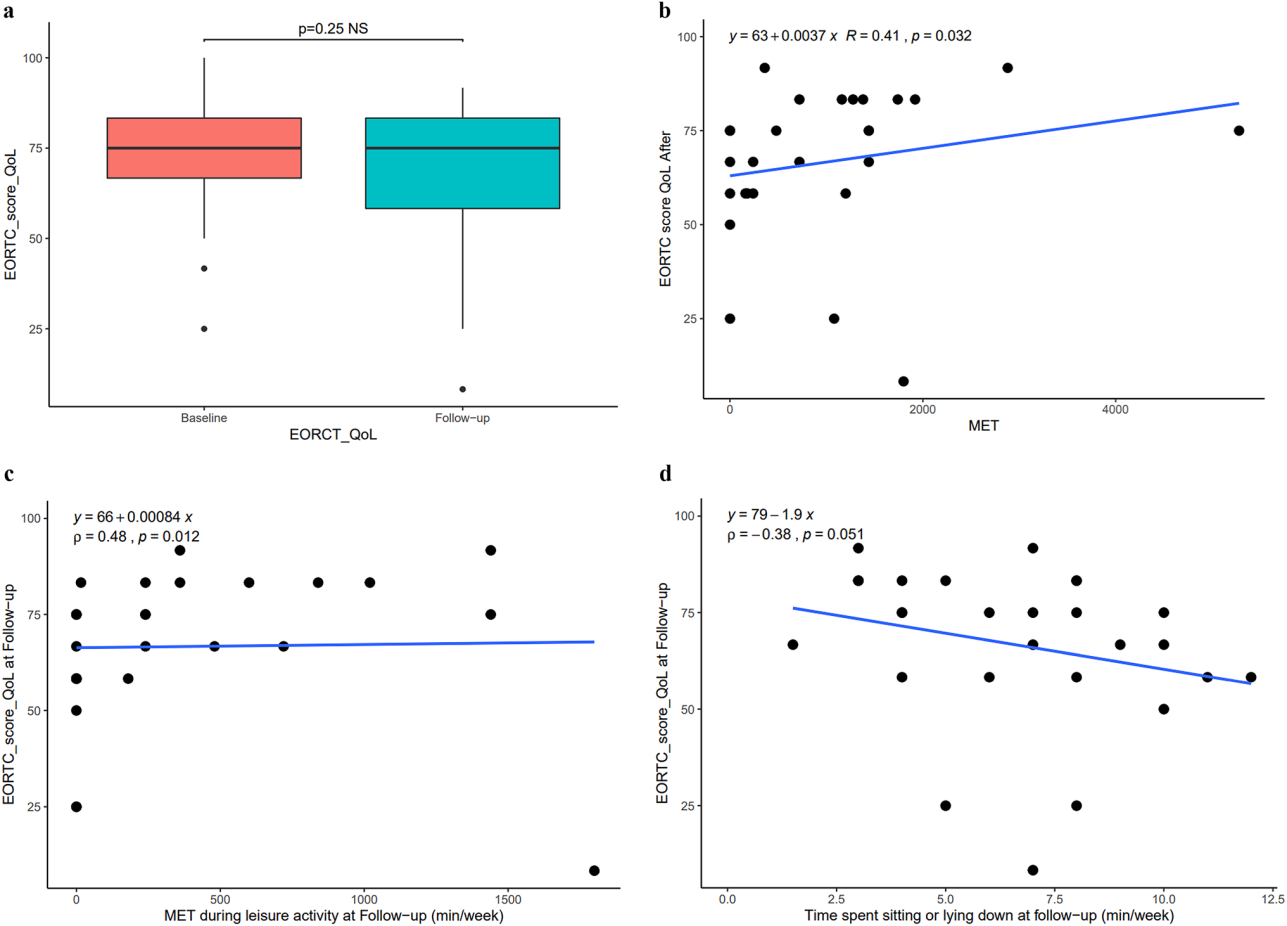
**a** No significant difference in quality of life (ETORC QoL) between interventional evaluation before (baseline) and after lockdown (follow-up). **b** Evaluation of quality of life (ETORC score QoL) after lockdown (interventional evaluation follow-up) according to the amount of energy expended on a weekly basis (baseline). **c** Evaluation of quality of life (EORTC QoL) according to the amount of energy (MET min/week) expended on a weekly basis during leisure activities. **d** Evaluation of quality of life (ETORC QoL) according to the amount of time spent sitting or lying down during lockdown (h/day**)**

No significant difference in overall health status (QoL) was shown post-lockdown based on the EORTC QLQ-C30 questionnaire (Fig. 2a). However, an increase, i.e. significant improvement in social interactions was recorded on the functional scale (*p* = 0.008).

According to the Spearman test, a high total energy expenditure during lockdown was significantly correlated with a better quality of life post-lockdown (*p* = 0.03; rho = 0.4133) (Fig. 2b).

Similarly, after the lockdown, subjects who had expended high levels of energy in leisure activities enjoyed a better quality of life (*p* = 0.01; rho = 0.4751) (Fig. 2c).

Finally, a poorer quality of life post-lockdown was correlated with prolonged sitting or lying down during the lockdown (*p* = 0.05; rho = − 0.1927) (Fig. 2d).

#### Secondary objectives

o *Psychosocial assessment*

No change in psychological status was highlighted in the Kessler test between the two assessment periods.

**Fig. 3:**
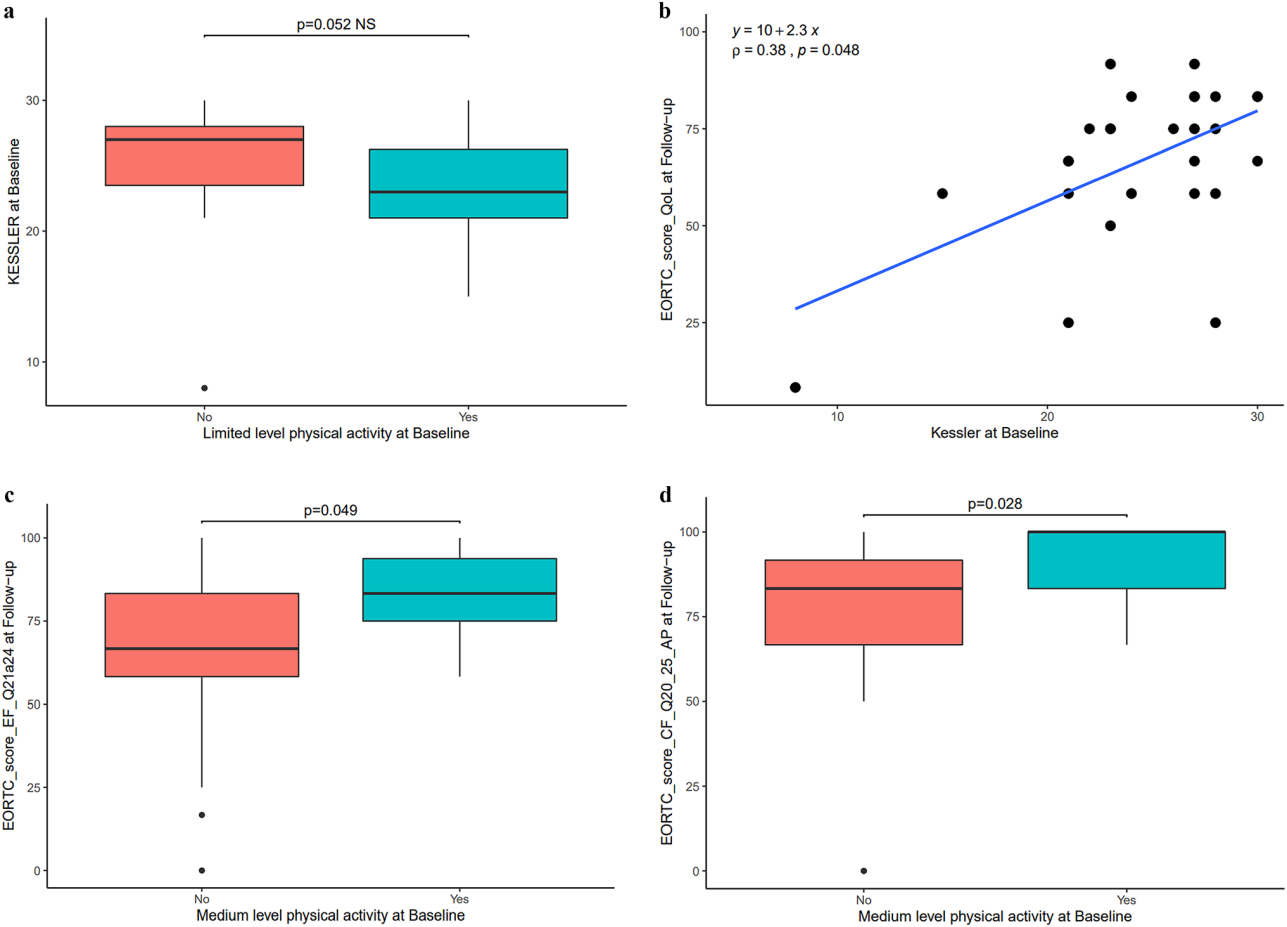
**a** Kessler score correlated to the intensity of physical activity. Limited level physical activity at Baseline—low-level physical activity at Baseline. **b** Quality of life after lockdown (follow-up) correlated to psychological status prior to lockdown (baseline). **c** Emotional functioning score at follow-up (EF Q21a24) correlated to the mean overall activity at baseline (before lockdown). Medium level physical activity at Baseline—moderate-level physical activity at Baseline. **d** Cognitive functioning score at follow-up (CF Q20 25) correlated to mean overall activity at baseline. Medium level physical activity at Baseline—moderate-level physical activity at Baseline

However, during the lockdown, participants with a low level of activity had a higher score on the Kessler scale, indicating a higher level of psychological distress (p = 0.05; rho = − 0.3772) (Fig. 3a). It is also important to note that when the level of psychological distress before lockdown was high, the quality of life after lockdown was poorer (p = 0.04; rho = 0.3835) (Fig. 3b).

On the other hand, there is a correlation between high-energy expenditure before the lockdown and a higher EF score after lockdown, i.e. improved emotional functioning (p = 0.04; rho = 0.3822) (Fig. 3c).

In addition, a higher CF score after lockdown is correlated to average activity before the lockdown, i.e. better cognitive functioning when the activity level was at least average (p = 0.02; rho = 0.4234) (Fig. 3d). On the other hand, there was no correlation between the level of physical activity after the lockdown and psychological functioning.

o Assessment of symptoms

There were no significant differences in pre- and post-lockdown symptoms.

A correlation was noted between perceived post-lockdown fatigue and the level of physical activity prior to lockdown. The higher the average total activity level, the lower the level of fatigue (*p* = 0.009). Similarly, the higher the level of fatigue after lockdown, the poorer the quality of life (*p* = 0.005).

With regards to sleep disorders and “insomnia”, increased post-lockdown insomnia was related to the time spent sitting or lying down during the lockdown (*p* = 0.01; rho = 0.4481) and was correlated to more psychological distress (*p* = 0.01; rho = 0.4486) and a lower quality of life score (i = 0.02; rho = 0.4270).

Post-lockdown “pain” was not correlated with the level of physical activity. On the other hand, a high level of pre-lockdown pain was correlated with a lower level of post-lockdown physical activity.

There was also no correlation between “dyspnoea” and the level of physical activity.

## Discussion

Multiple studies have shown that APA can reduce the risk of mortality in cancer patients, particularly with regard to breast cancer. A meta-analysis reported that physical activity post-diagnosis was associated with a 34% reduction in the risk of specific mortality, a 41% reduction in the risk of overall mortality and a 24% reduction in the risk of recurrence (Ibrahim and Al-Homaidh 2011; Schmid and Leitzmann 2014). The World Health Organization has launched numerous national plans to encourage cancer patients to engage in regular physical activity for at least 150 min per week (Kushi et al. 2010). Many specific assessment programmes are currently offered as soon as patients are admitted to health care facilities (Bouillet et al. 2015). Since this approach must be sustained for at least 18 months, sports associations affiliated to sports federations are setting the scene by offering a wide range of activities such as fencing, tennis, walking, dance, gymnastics and karate in a specific setting (Leroy 2017; Short et al. 2015; Loo et al. 2019).

In addition, the French Cancer Institute (INCA) and the French Health Authority (HAS) have issued recommendations to improve patient motivation and to reduce barriers of a personal, cultural, organisational and environmental nature that impede the practice of adapted physical activity from the moment cancer is diagnosed (Bénéfices de l’activité physique pendant et après cancer 2017). It is in this context that we wished to offer a range of adapted physical activities, including rugby, as soon as the diagnosis of cancer is announced. Rugby is a group activity with values of support and solidarity. As Hardcastle et al. have shown, cancer survivors lose confidence in their physical ability (Hardcastle et al. 2017). Therefore, according to the strategy developed by Avancini et al. (2020b) to improve the adherence and compliance of participants, we think it is important to offer playful, flexible activities supervised by a specialised sport educator, taking into account the wishes of the participant, and their previous experiences and environment. All information concerning the expected benefits of the practice are shared at the beginning of treatment. Finally, patients are regularly assessed to encourage them to continue their efforts (Bouillet et al. 2015).

Many physical activity rehabilitation programmes for cancer survivors have been evaluated and shown to be beneficial for treatment tolerability, quality of life and/or disease prognosis. However, no study has been able to show the effects of stopping one of these programmes abruptly. Total lockdown, both in France and worldwide, is unprecedented. Literature on the impact of suddenly stopping physical activity is sparse, with even less information on people with chronic diseases (Pietrobelli et al. 2020; Mukhtar 2019; Bonora et al. 2020).

Our aim was to assess the impact of abruptly stopping an adapted supervised physical activity, such as rugby for health, in a cohort of cancer patients.

Our series focuses on cancer patients who had been playing rugby for health on a regular basis for at least one season. Several physical activities are suggested to patients during consultation, including rugby. They are supervised by APA and rugby instructors.

The first question in the national cross-sectional survey during the last 7 days of the French lockdown asked participants how they felt about the sudden discontinuation of their main physical activity. The participation rate of 87.5% is very high compared to the literature. This can be attributed to the fact that participants are aware of the general well-being provided by this group activity and of the need for a post-lockdown assessment before activity can resume (Lee et al. 2020).

Overall, the survey tells us that almost 50% of the participants experienced a moderate to severe impact on sleep, anxiety, general well-being, treatment tolerability and general health. Participants also reported a weight gain of 3–5 kg with a significant increase in weight and hip circumference noted in the individual analysis. We found similar significant results in the interventional evaluation. In an Italian cohort of 41 children, Pietrobelli et al. (2020) highlighted the negative impact of the COVID-19 lockdown on the daily behaviour of obese children. Weight gain in obese children was due to changes in eating behaviour, interrupted sleep patterns and reduced activity.

To get a grasp on the adapted way to resume physical activity, we believe it is important to know how patients experienced this period. At the same time, we also thought it would be important to continue the investigations with more objective data. This is why we proposed an interventional evaluation. To our knowledge, there is no similar report in the literature.

Results from a cross-sectional study on 4005 individuals in France showed that more than 8 in 10 respondents reported unhealthy changes in lifestyle since the COVID-19 lockdown, most of which were common during this period, and which especially concerned physical activity [ref]. On the other hand, the analysis conducted by Lee et al. (2020) showed that post-cancer APA patients were more likely to participate in leisure-type physical activity than those without the disease. Post-cancer participants appear to be far more aware of the benefits of physical activity. In our survey, 75% of the participants reported maintaining regular physical activity but the interventional analysis reported a significant decrease in the intensity of physical activity.

Our study showed no impact of the lockdown on quality of life and psychological status despite the significant decrease in intense physical activity. The same applies to the number of hours spent sitting or lying down, which significantly increased during the lockdown.

However, a significant correlation was noted between a high level of total energy expenditure during the lockdown and the quality of life. A high level of total energy expenditure during the lockdown is correlated to an improved quality of life during this period. Similarly, the lower the activity level, the higher the Kessler score, reflecting a higher level of psychological distress.

To our knowledge, no comparable evaluation has been published regarding cancer patients who participate in adapted physical activity for health improvement. An Italian study that evaluated the impact of reduced physical activity during the lockdown on patients with neuromuscular disorders highlighted a change in the quality of life related to the level of weekly physical activity (Stefano et al. 2020). Another study proposed an interesting model of a home-based exercise dedicated to cancer patients in the COVID-19 era (Avancini et al. 2020a).

The limitations of our study must be considered. First, they mainly concern the size of the cohort and the lack of comparison with healthy participants. In this unprecedented context, it was difficult to consider a comparative study between two populations, but a repeat assessment will be conducted a few weeks post-lockdown. Second, the cross-sectional design does not allow causal inferences about relationships between variables to be drawn.

There is already evidence to show that physical activity improves mental health in healthy individuals (Jiménez-Pavón et al. 2020). In our study, the higher the energy expenditure before lockdown, the better the EF score in the post-lockdown period, which reflects improved emotional functioning.

In conclusion, our study showed no impact of the lockdown on the overall health and psychological status of cancer patients who regularly participate in rugby for health. On the other hand, we highlighted a negative impact on the level of physical activity with reduced intensity across the board. We found a correlation between a decrease in intensity and quality of life and psychological distress.

Our findings suggest that the unifying nature of “rugby for health”, a lockdown limited to 2 months, regular practice before lockdown, and sustaining a substantial level of physical activity during lockdown helped participants to maintain a satisfactory quality of life and mental stability. Benefits of physical activity for cancer survivors should be considered in the context of prolonged COVID pandemics.

## Supplementary Information

Below is the link to the electronic supplementary material.

Supplementary file1 (DOCX 20 kb)

## Data Availability

RUBIES Association Rugby Bien être Santé.
