# Supplementary material for: Evaluation of the impact of the COVID-19 lockdown on the quality of life of patients monitored for cancer who practice an adapted physical activity: rugby for health
Source: J Cancer Res Clin Oncol. 2021 Apr 5;148(2):425–39. doi: 10.1007/s00432-021-03621-7 (PMC8021215; doi:10.1007/s00432-021-03621-7)
Supplement: Supplementary file 1 — Supplementary file1 (DOCX 20 kb) [file 432_2021_3621_MOESM1_ESM.docx]

APPENDIX 1: 42-question survey to assess the experience of participants in rugby for health during the first lockdown

| Question 1 | How old are you? | - Less than 30 years old - 30 to 60 years old - More than 60 years old |
| --- | --- | --- |
| Question 2 | What is your gender? | - Female - Male |
| Question 3 | In which town do you live? | Free response |
| Question 4 | Do you smoke? | - Yes - No |
| Question 5 | If so, how many cigarettes do you smoke per day? | - Less than 10 - More than 10 |
| Question 6 | If so, how long have you been smoking (in years)? | - 0 - Less than 10 years - More than 10 years - More than 20 years |
| Question 7 | For which cancer have you been you treated? | - Breast - Gynaecological - Digestive - Haematological - Prostate - Kidney - Lung - Other |
| Question 8 | How long ago? | - Less than one year - 1 to 3 years - 3 to 5 years - More than 5 years |
| Question 9 | Which treatment(s) did you receive? | - Surgery - Chemotherapy - Radiotherapy - Hormone therapy - Radio frequencies - Other |
| Question 10 | Do you have any ongoing treatments? | - No - Chemotherapy - Radiotherapy - Hormone therapy - Other |
| Question 11 | Have you had a recurrence of cancer? | - Yes - No |
| Question 12 | To your knowledge, have you had a COVID-19 infection? | - No - Yes, positive test without hospitalisation - Yes, positive test or scan with hospitalisation - Yes, positive test or scan with admission to intensive care |
| Question 13 | Before the health crisis, how long had you been playing rugby for health? | - Less than one season - More than one season - More than 2 seasons - More than 3 seasons |
| Question 14 | Did you play any sport in the 5 years before you started playing rugby for health? | - Yes - No |
| Question 15 | Before the health crisis, did you play any other sport (besides rugby for health)? | - Yes - No |
| Question 16 | If so, how many? | - One other sport - Two others - Three others, or more |
| Question 17 | If so, which ones? | Free response |
| Question 18 | Did you start any other sport after you started rugby health? | - Yes - No |
| Question 19 | If you used to smoke, did you stop smoking after starting rugby for health? | - No - Yes, permanently - Yes, but restarted - Not applicable |
| Question 20 | Before the health crisis, on an average, how many sessions of rugby for health did you participate in per week? | - One session - 2 sessions - 3 or more |
| Question 21 | Since the lockdown, have you continued a physical activity at home? | - Yes - No |
| Question 22 | If so, which one? | Free response |
| Question 23 | How many times a week? | - 0 - 1 to 2 times - 3 to 4 times - Every day of the week (at least 5 times) |
| Question 24 | How long does an average session last? | - 0 - Approximately 15 minutes - Approximately 30 minutes - 1 hour or more |
| Question 25 | Since the lockdown, how long do you walk per day? | - Less than 30 minutes - More than 30 minutes - More than an hour |
| Question 26 | In your opinion, since the start of the lockdown, has your energy expenditure increased or decreased? | - Increased - Decreased - Stable |
| Question 27 | Have you gained weight since the lockdown? | - No - Yes - I don't know |
| Question 28 | How much? | - 0 - Less than 5 kg - 5 to 10 kg - More than 10 kg |
| Question 29 | Have you started smoking again since the lockdown? | - No - Yes - Not applicable |
| Question 30 | During the lockdown, did you work? | - Yes, at the workplace - Yes, I teleworked - No |
| Question 31 | Did you change your mode of transport during the lockdown? | - Yes - No |
| Question 32 | If so, what were the changes? | Free response |
| Question 33 | Before the crisis (lockdown), how much time per day did you spend in front of a screen (smartphone, tablet, TV, etc.)? | - Less than 3 hours - 3 to 6 hours - More than 6 hours |
| Question 34 | Since the crisis (lockdown), how much time per day do you spend in front of a screen (smartphone, tablet, TV, etc.) ? | - Less than 3 hours - 3 to 6 hours - More than 6 hours |
| Question 35 | Since the lockdown, have you kept in touch with other participants in rugby for health? | - No - Yes, by phone from time to time - Yes, often by phone - Yes, by video calls from time to time - Yes, by video calls often |
| Question 36 | Since the lockdown, have you had difficulty sleeping? On a scale of 0 to 10 (0 - none, 10 - very significant), how would you describe it? | On a scale of 0 to 10 |
| Question 37 | Since the lockdown, have you been anxious or worried? On a scale of 0 to 10 (0 - no, 10 - very significantly), how would you describe it? | On a scale of 0 to 10 |
| Question 38 | Since the lockdown, have you experienced any new joint or muscle pain? On a scale of 0 to 10 (0 - none, 10 - very significant), how would you describe them? | On a scale of 0 to 10 |
| Question 39 | Since the lockdown, have you felt a decline in your general health (fitness)? On a scale of 0 to 10 (0 - none, 10 - very significant), how would you describe it? | On a scale of 0 to 10 |
| Question 40 | Do you think that the lockdown and the cessation of supervised physical activities have had an impact on the tolerability of your past or current treatments? On a scale of 0 to 10 (0 - none, 10 - very significant), how would you describe it? | On a scale of 0 to 10 |
| Question 41 | Do you think that the lockdown and the cessation of supervised physical activities have had an impact on your health? On a scale of 0 to 10 (0 - none, 10 - very significant), how would you describe it? | On a scale of 0 to 10 |
| Question 42 | Do you plan to resume your usual physical activities as soon as possible? | - Yes - No - Perhaps |
